# Expansion of Human Pluripotent Stem Cell-derived Early Cardiovascular Progenitor Cells by a Cocktail of Signaling Factors

**DOI:** 10.1038/s41598-019-52516-8

**Published:** 2019-11-05

**Authors:** Sadaf Vahdat, Sara Pahlavan, Elena Mahmoudi, Maryam Barekat, Hassan Ansari, Behnaz Bakhshandeh, Nasser Aghdami, Hossein Baharvand

**Affiliations:** 1 Department of Stem Cells and Developmental Biology, Cell Science Research Center, Royan Institute for Stem Cell Biology and Technology, ACECR, Tehran, Iran; 2 Department of Biotechnology, College of Science, University of Tehran, Tehran, Iran; 3 Department of Regenerative Medicine, Cell Science Research Center, Royan Institute for Stem Cell Biology and Technology, ACECR, Tehran, Iran; 4 Department of Developmental Biology, University of Science and Culture, Tehran, Iran

**Keywords:** Multipotent stem cells, Stem-cell research

## Abstract

Cardiovascular progenitor cells (CPCs) derived from human pluripotent stem cells (hPSCs) are proposed to be invaluable cell sources for experimental and clinical studies. This wide range of applications necessitates large-scale production of CPCs in an *in vitro* culture system, which enables both expansion and maintenance of these cells. In this study, we aimed to develop a defined and efficient culture medium that uses signaling factors for large-scale expansion of early CPCs, called cardiogenic mesodermal cells (CMCs), which were derived from hPSCs. Chemical screening resulted in a medium that contained a reproducible combination of three factors (A83-01, bFGF, and CHIR99021) that generated 10^14^ CMCs after 10 passages without the propensity for tumorigenicity. Expanded CMCs retained their gene expression pattern, chromosomal stability, and differentiation tendency through several passages and showed both the safety and possible cardio-protective potentials when transplanted into the infarcted rat myocardium. These CMCs were efficiently cryopreserved for an extended period of time. This culture medium could be used for both adherent and suspension culture conditions, for which the latter is required for large-scale CMC production. Taken together, hPSC-derived CMCs exhibited self-renewal capacity in our simple, reproducible, and defined medium. These cells might ultimately be potential, promising cell sources for cardiovascular studies.

## Introduction

Cardiovascular diseases, especially ischemic heart conditions, are considered one of the leading causes of death worldwide. Numerous studies have been conducted to develop novel strategies to increase both patients’ quality of life and longevity^1^. Despite these efforts, no effective treatment exists for these patients^2^. Recently, cardiac cell therapy has become a promising approach and may replace commonly used treatments. In this regard, researchers propose the use of different cell sources, including those which have been isolated from both non-cardiac and cardiac origins^3^. Among these various cell sources, adult stem cells isolated from non-cardiac origins such as bone marrow-derived stem cells, could not efficiently differentiate into three major cardiac lineages and their regenerative benefits for cardiovascular diseases remain controversial^4^. However, the majority of these cells appear to be clinically safe and their positive impacts on heart performance are mostly attributed to paracrine effects.

Over the last decade, global attention has turned to cardiac lineage cells such as cardiomyocytes and cardiovascular progenitor cells (CPCs) as promising cell sources for cardiac cell therapy. However, the challenges of *in vitro* culture and the electrical coupling to the host myocardium restrict the application of cardiomyocytes in clinical studies^3^. Therefore, this new area may benefit from a cardiac-committed, preferably autologous cell type that has the capability for *in vitro* expansion, engraftment after transplantation, and differentiation into cardiovascular lineages *in vivo*^5,6^. Development of an *in vitro* culture system for large-scale production and long-term maintenance of cardiac cell types, especially progenitor cells, is in tremendous demand^5^.

CPCs can be generated by *in vitro* differentiation of human pluripotent stem cells (hPSCs) into cardiovascular lineages. CPCs are clonogenic and have self-renewal capacity as well as the ability to differentiate into cardiac lineages^5,7^. Until now, hPSC-derived CPCs (SSEA1^+^ cells) were used in rodents after myocardial infarction (MI)^8^, non-human primates models^9,10^ and human clinical trials^11^, which suggests that they may be promising sources for cardiac regenerative medicine. Moreover, cardiogenic mesodermal cells (CMCs), which are early CPCs, may hold great promise for cardiac regenerative medicine^12,13^. CMCs are characterized by the expression of mesoderm posterior 1 (MESP1) transcription factor and can differentiate into almost all cardiac cell types both *in vitro* and *in vivo*^13–17^. Therefore, the field of cardiac cell therapy can benefit from the establishment of a culture system for maintenance and long-term/large-scale expansion of CMCs^5^.

In the current study, we aimed to introduce a defined, simple, and reproducible protocol for long-term expansion, maintenance, and storage of hPSC-derived CMCs. We screened some cell signaling factors to develop a chemically defined culture medium. Cultured CMCs expanded for more than 10 passages resulting in >10^14^ cells, and retained their morphology, molecular pattern, and *in vitro* differentiation propensity into cardiac lineages over passages. We observed no tumorigenicity, engraftment of the self-renewed cells, and improved cardiac performance after transplantation into rat ischemic hearts. This culture condition is reproducible and capable of transformation to a carrier-free suspension culture, which is required for large-scale production of CMCs. Our results provide a novel approach for long-term self-renewal and maintenance of early CPCs, which is a fundamental step for commercialization, developmental, tissue engineering, and cell-based clinical studies.

## Results

### ***In vitro*** generation of CMCs

**Figure 1:**
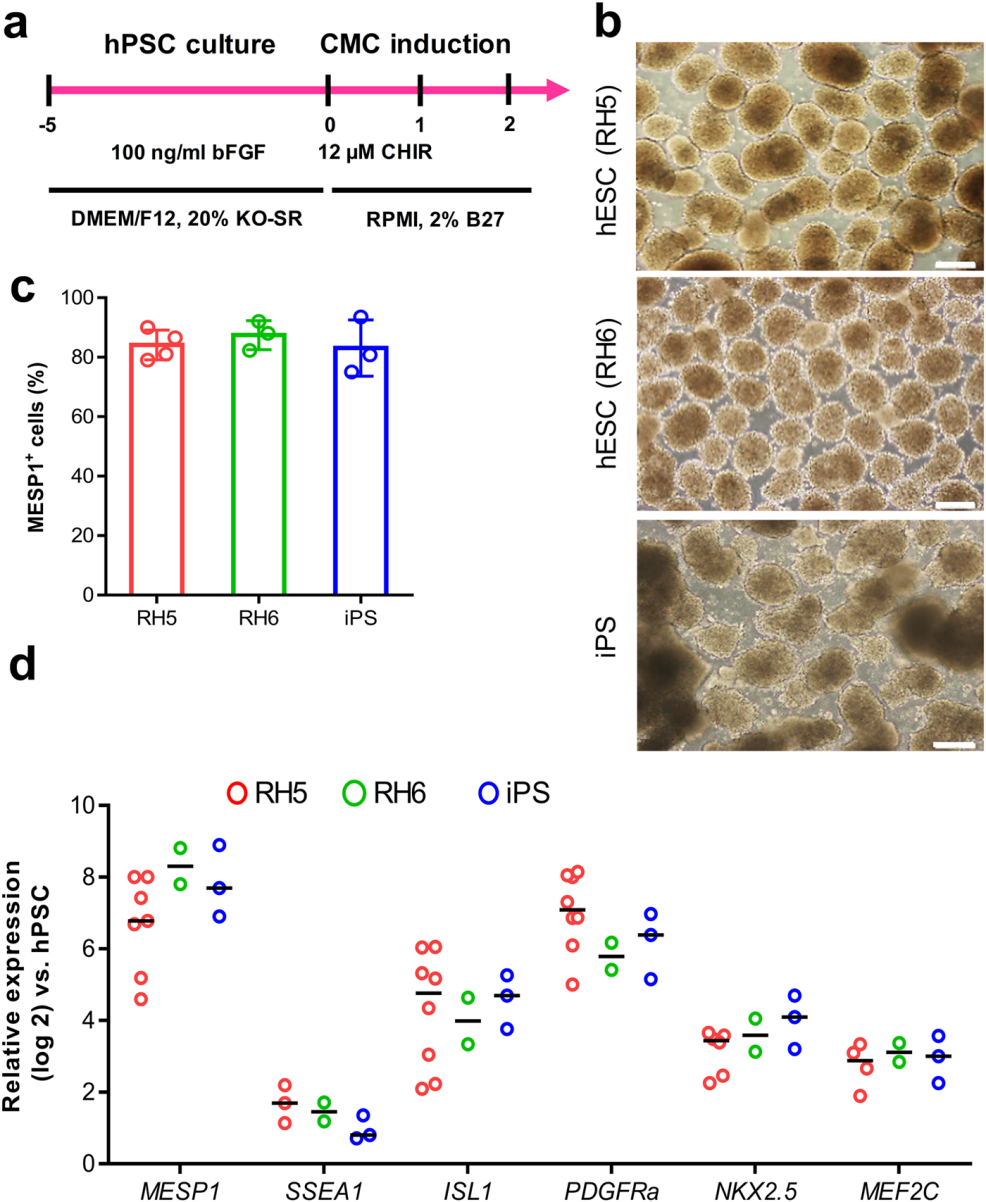
Characterization of CMCs generated from three hPSC lines. (**a**) Schematic diagram showing differentiation protocol used for CMC induction from hPSCs. hPSC lines were cultured in suspension as spheroids and differentiated into mesendoderm, followed by cardiac mesoderm lineages by one-day treatment with 12 µM CHIR99021 (CHIR) and a one-day rest period. (**b**) Morphology of the CMC spheroids derived from two hESC lines (RH5 and RH6) and an iPS line. Scale bars: 200 µm. (**c**) Percentages of MESP1^+^ cells in CMC spheroids derived from RH5, RH6, and iPS cells. Data: mean ± standard deviation (SD). (**d**) Expression analysis of cardiac mesodermal and cardiac-specific genes in CMC spheroids. Data presented as median.

A suspension culture system was used to expand and differentiate two human embryonic stem cell (hESC) lines (RH5, RH6) and one hiPSC line (iPS) into CMCs as previously described (Fig. 1a,b)^12,18^. Flow cytometry analysis showed that 83.3 ± 5.8% of the RH5-CMCs were MESP1^+^. Furthermore, 87.4 ± 5.0% MESP1^+^ cells were identified in RH6-CMCs and 83.1 ± 9.5% in the iPS-CMCs (Fig. 1c). CMC spheroids, generated from all three pluripotent cell lines, expressed cardiac mesoderm markers and cardiac-specific transcription factors *MESP1*, *SSEA1*, *ISL1*, *PDGFRa*, *NKX2.5*, and *MEF2c* (Fig. 1d).

### Screening of signaling pathway factors for long-term expansion of CMCs

**Figure 2:**
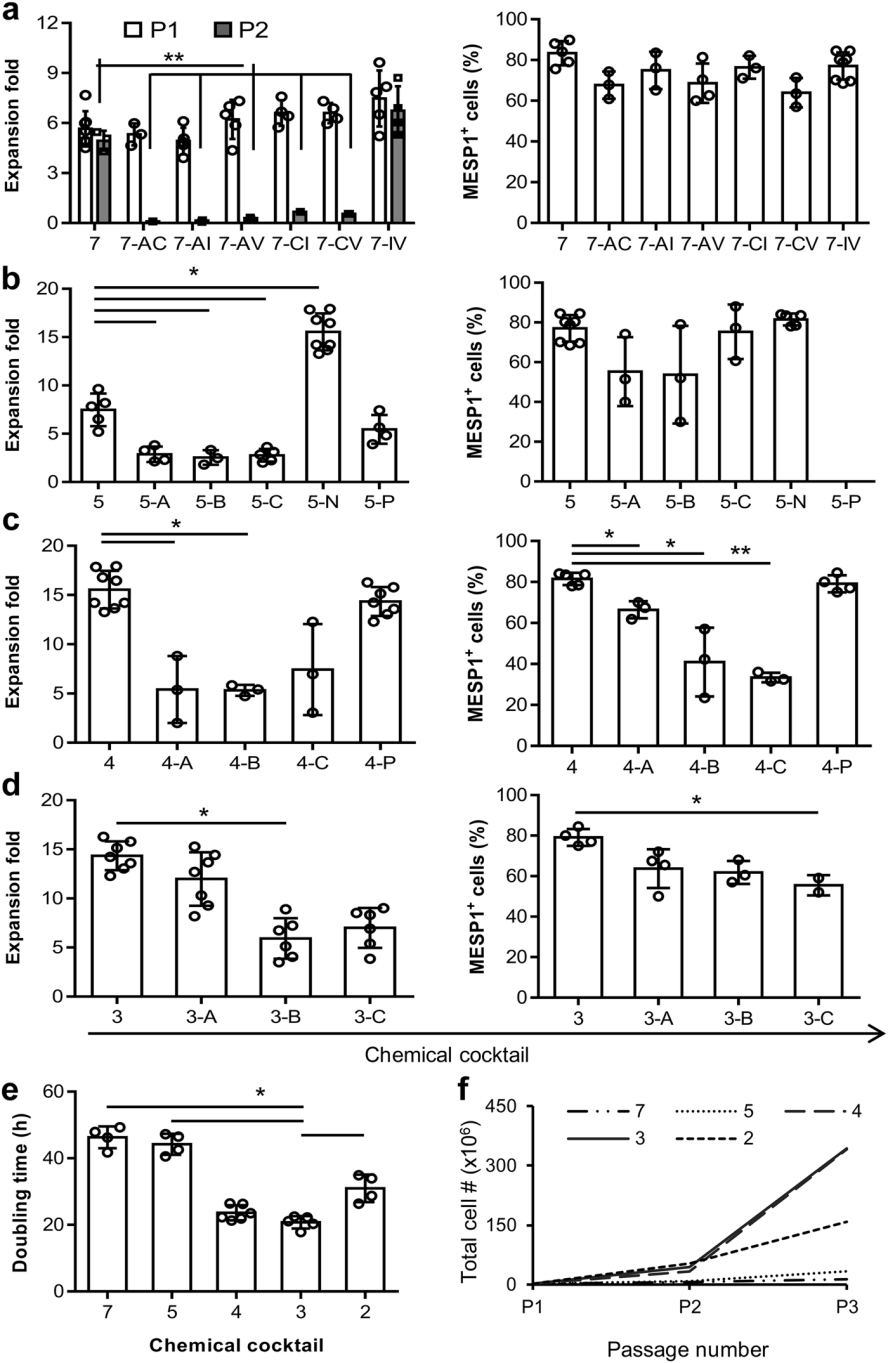
A top-down chemical screening to find the most efficient combination of signaling factors for self-renewal of CMCs under adherent culture conditions. **(a**–**d**) The expansion fold and percentage of MESP1^+^ cells were evaluated as two preliminary criteria to find the minimal essential pool of signaling molecules. In each run, the effect of different combinations of n-1 signaling factors in culture media was assessed according to these criteria. Serial removal of factors resulted in the selection of 7-IV (5), 5-N (4), 4-P (3) and 3-A (2). “–”: Indicates withdrawal of signaling factors. A: A83-01; B: bFGF; C: CHIR; I: IGF1; N: NRG1 agonist; P: Pioglitazone; V: Vitamin C; P1: passage 1; P2: passage 2. (**e**) The doubling time of CMCs in culture media that contained selected combinations of factors. The 3 and 4 combinations of signaling chemicals had the least doubling time of the CMCs. (**f**) The total cell count of CMCs after three passages in media that contained the selected factors. As presented, CMCs had the highest cell count in media that contained the 3 and 4 combinations of chemicals. All data: mean ± SD and statistically analyzed by one-way ANOVA followed by Tukey’s post-hoc test. *P ≤ 0.05; **P ≤ 0.01; ^#^Number.

In order to find an efficient defined medium for prolonged culture and self-renewal of CMCs, based on the literature, we selected eight factors that had putative proliferation potential (Table 1). A top-down approach was designed to find the most effective cocktail of factors. RH5-CMC spheroids were dissociated into single cells and cultured adherently in the presence of selected chemicals. The percentage of MESP1^+^ cells and the expansion fold were assessed as the two criteria for the cocktail selection (Fig. 2a–d). Initially, different combinations of 8-1 factors (all factors minus one), were used which resulted in the CMCs expansion in 8-D (all factors minus dorsomorphin, named as 7) (Supplementary Fig. S1). Therefore, the 8-D medium was chosen for the next top-down step. Further removal of one chemical from 8-D resulted in a substantial decrease in cell proliferation. The top-down approach was followed by investigating the impact of removing two factors from 7 factor mixture (Fig. 2a). While there were no significant differences between the 7-2 media groups at the first passage, the CMCs showed the highest expansion fold in the absence of IGF1 and ascorbic acid (7-IV, named as 5 in the following steps) at the second passage (Fig. 2a and Supplementary Fig. S1). Serial removal of signaling factors resulted in the selection of the 5-N (4), 4-P (3), and 3-A (2) combinations based on proliferation rate and preservation of the MESP1^+^ cell population in the subsequent steps (Fig. 2b–d). To achieve the most efficient cocktail with the least combination of chemicals, 7, 5, 4, 3, and 2 media were compared for population doubling time (PDT) and cumulative total cell count (Fig. 2e,f). The cocktail of 4 and 3 factors had the least PDT (Fig. 2e). Accordingly, 4 and 3 media had the highest cell count after three passages (Fig. 2f). Furthermore, when CMCs were cultured in medium that contained only one chemical, the medium that contained either A83-01, bFGF or CHIR99021 (CHIR) showed larger fold changes of expansion compared to the other five factors (Supplementary Fig. S2). Therefore, three factors cocktail that contained A83-01, bFGF, and CHIR was selected as the most efficient expansion medium, which was denoted as ABC medium.

**Table 1:** Candidate signaling factors examined for maintenance and expansion of CMCs.

Candidate factor (abbreviation)	Activity	Reference
A83-01 (A)	Activin/nodal inhibitor	^19, 26^
bFGF (B)	FGF signaling activator	^27^
CHIR99021 (C)	Wnt signaling activator	^19^
Dorsomorphin (D)	BMP signaling inhibitor	^19^
IGF1 (I)	Activation and phosphorylation of Akt and mTOR	^28^
NRG1 agonist (N)	Potentiating the NRG1/ERBB4 signaling pathway	^29^
Pioglitazone (P)	PPARγ receptor activator	^30^
Vitamin C (V)	Chromatin modulator, MEK-ERK1/2 signaling modulator	^31^

Then, different concentrations of A83-01 (0.5, 1, and 2 µM), bFGF (10, 25, 50, and 100 ng/ml), and CHIR (3, 6, and 12 µM) were tested to obtain the optimal concentrations based on the CMC proliferation rate (Supplementary Fig. S3). The increasing A83-01 concentration led to a substantial reduction in cell count. In contrast, higher concentrations of bFGF (100 ng/ml) resulted in increased cell numbers. Similar to A83-01, increasing the CHIR concentration resulted in a significant reduction in cell expansion. These results suggested that 0.5 µM A83-01, 100 ng/ml bFGF, and 3 µM CHIR (ABC medium) could be the optimal concentrations for expansion of CMCs.

### Preservation of CMC characteristics after passages

**Figure 3:**
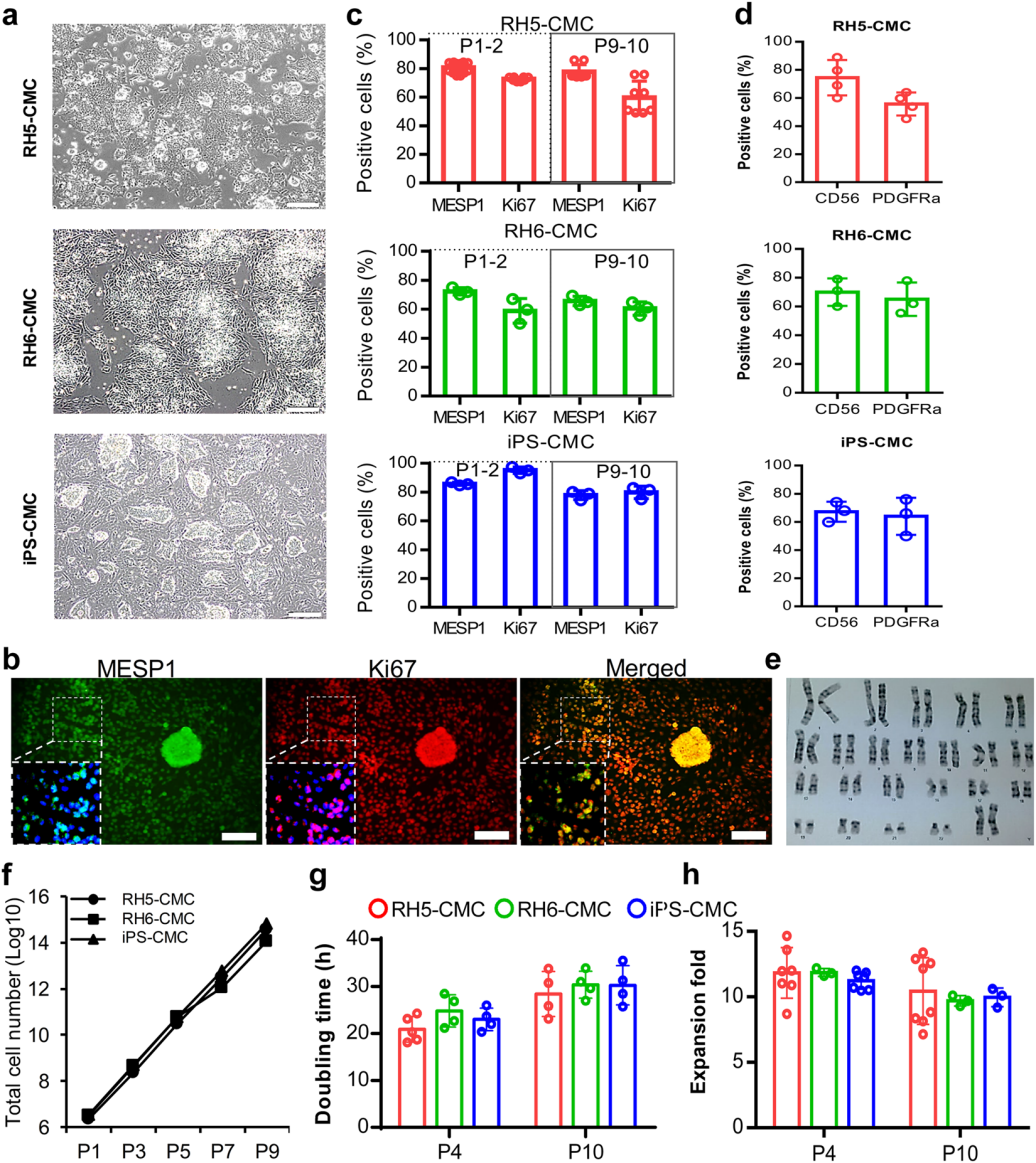
Characterization of CMCs after passages under adherent culture conditions in ABC medium. (**a**) Morphology of cultured CMCs derived from RH5, RH6, and iPS lines in ABC medium. (**b**) Immunofluorescence analysis of MESP1 and Ki67 expressions in CMCs cultured in ABC medium. Cells were counter-stained with DAPI. (**c**) Representative graphs show the percentages of MESP1^+^ and Ki67^+^ cells in cultured CMCs derived from RH5, RH6, and iPS lines at early (1–2) and late (9–10) passages as analyzed by flow cytometry. (**d**) PDGFRα and CD56 surface marker analysis of CMCs at the late passages. (**e**) Karyotype image of cultured RH5-derived CMCs in ABC medium at passage 10 (P10) that shows the normal cell karyotype. (**f**) Representative graph of total cell counts generated after nine passages of RH5-, RH6- and iPS-derived CMCs in ABC medium. (**g**,**h**) Doubling time and expansion fold of CMCs at early and late passages. Scale bars: 200 µm. All data: mean ± SD. The statistical differences between P4 and P10 in all experimental groups were analyzed by unpaired t-test.

**Figure 4:**
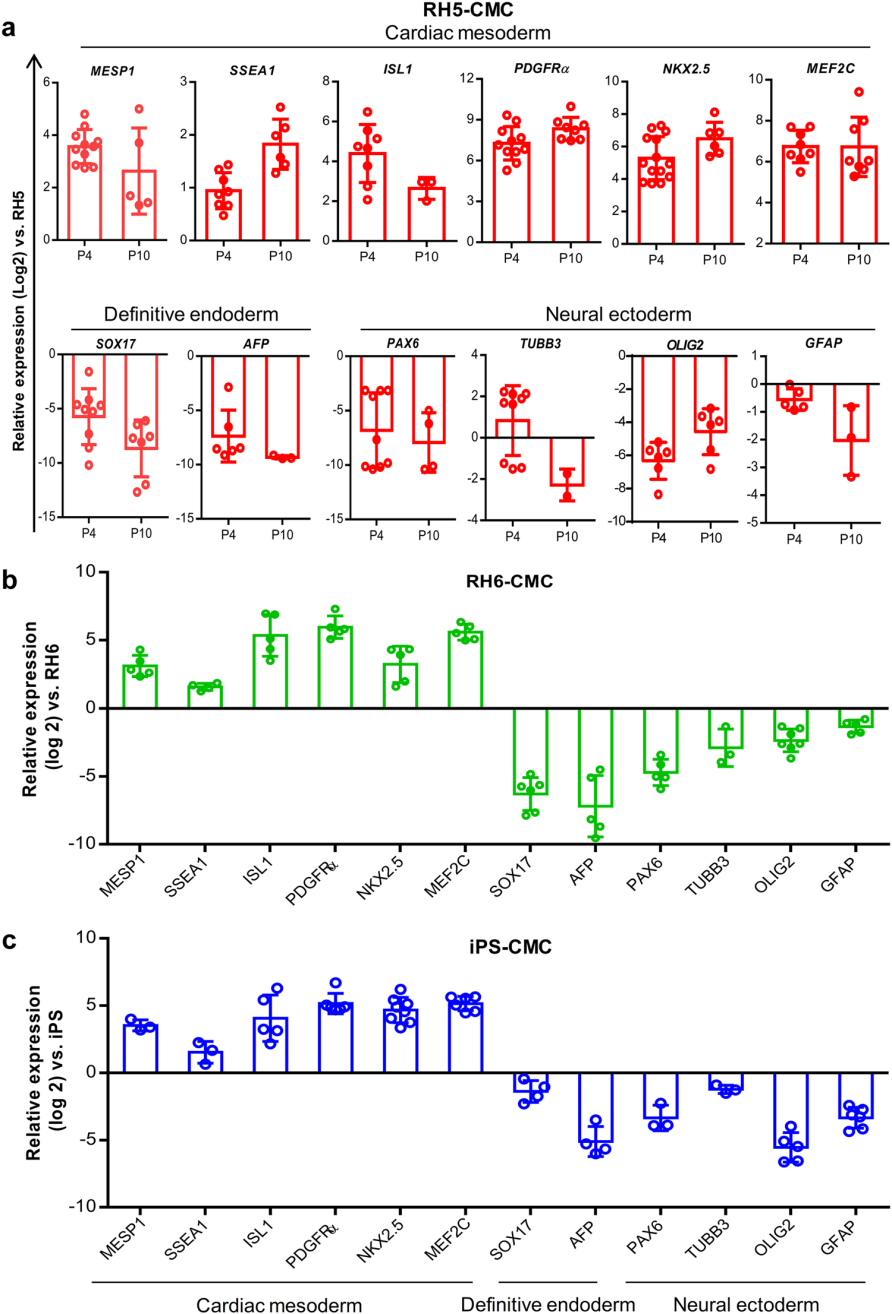
Expression pattern of cardiac mesoderm, definitive endoderm, and neural ectoderm genes in adherently expanded CMCs. Quantitative RT-PCR analysis showed upregulation of cardiac mesoderm and downregulation of definitive endoderm and neural ectoderm genes in CMCs derived from RH5 (**a**), RH6 (**b**), and iPS (**c**) cell lines. Analysis was performed at passage 4 (P4) for (**b**) and (**c**). All data: mean ± SD. The statistical differences between P4 and P10 in (**a**) were analyzed by the unpaired t-test.

Cultured CMCs in ABC medium showed epithelial-like morphology with co-expressions of MESP1 and Ki67, and generated colonies that had a dome-like appearance (Fig. 3a,b). In order to determine whether cultivation in ABC medium could preserve CMC characteristics over passages, the percentages of MESP1^+^ and Ki67^+^ cells were assessed at the early (P1–2) and late (P9–10) passages. Flow cytometry results showed that RH5-CMCs were 80.8 ± 3.6% positive for MESP1 during the early passages and 77.7 ± 5.5% positive at the late passages. RH5-CMCs also had 72.7 ± 1.5% Ki67^+^ cells during the early passages and 59.7 ± 12.4% during the late passages (Fig. 3c). The same expressions of MESP1 and Ki67 were observed in CMCs derived from the RH6 and iPS lines at the early and late passages (Fig. 3c). The cultured cells expressed CD56 (a surface marker for mesodermal progenitors) and PDGFRα (a well-known CMC surface marker) (Fig. 3d). Moreover, the effect of extended proliferation was assessed on chromosome stability by performing cytogenetic analysis on P10 CMCs. The results showed that these CMCs had a normal karyotype (Fig. 3e). CMCs derived from the RH5, RH6, and iPS lines retained their proliferation rate for more than 9 passages, resulting in >10^14^ cells (Fig. 3f). Although the PDT and expansion fold showed slight differences over the passages, these findings did not statistically differ (Fig. 3g,h). Quantitative RT-PCR showed a similar expression pattern for CMC markers *MESP1*, *SSEA1*, and *PDGFRa*, as well as early CPC transcription factors *ISL1*, *NKX2.5*, and *MEF2c* among the passaged and P0 CMCs (Fig. 4a and Supplementary Fig. S4). However, the specific markers of definitive endoderm (*SOX17* and *AFP*) and neural ectoderm (*PAX6*, *TUBB3*, *OLIG2*, and *GFAP*) lineages were downregulated in the early and late passage CMCs (Fig. 4a and Supplementary Fig. S4). Similarly, cultivated RH6-CMC and iPS-CMC in ABC medium expressed cardiac mesoderm markers and downregulated the definitive endoderm and neural ectoderm genes (Fig. 4b,c).

In order to examine the tumorigenicity of the expanded cells in ABC medium, the early (P4), late (P10), and P0 CMCs were injected into nude mice. No sign of tumor formation was detected 90 days after the injections; however, transplantation of hPSCs led to the development of a recognizable tumor mass 20 days after the injections (Supplementary Table S2 and Supplementary Fig. S5). Taken together, these results indicated that the cells retained their original characteristics throughout passaging while the lack of tumorigenicity resolved their safety concerns.

### Cryopreservation and recovery of CMCs

**Figure 5:**
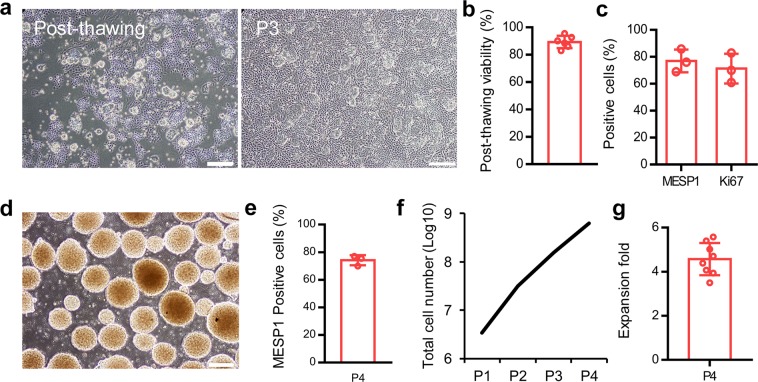
Long-term storage and suspension culture of CMCs. (**a**) The morphology of cryopreserved CMCs post-thaw and after three passages in ABC medium. (**b**) The survival rate of cryopreserved CMCs post-thaw. (**c**) The percentages of MESP1^+^ and Ki67^+^ cells in cryopreserved CMCs after three passages in ABC medium as assessed by flow cytometry. (**d**) The morphology of generated CMC-spheroids in ABC medium at day 4 of suspension culture. (**e**) The percentage of MESP1^+^ cells after four passages of CMCs in suspension culture as evaluated by flow cytometry. (**f**,**g**) The calculated total cell count and expansion fold after four passages of CMCs in suspension culture condition. Scale bars: 200 µm. All data: mean ± SD.

A simple and efficient freeze-thaw procedure was developed to store CMCs for extended periods of time and facilitate their transportation. Cryopreserved CMCs were grown in ABC medium (Fig. 5a). More than 90% of the cells were viable immediately after thawing (Fig. 5b). Furthermore, the percentages of MESP1^+^ and Ki67^+^ cells were retained, even after three passages post-recovery (Fig. 5c).

### Scalable suspension culture system of CMCs

In order to develop a scalable culture of CMCs, the adherent culture was transformed into a suspension system. Cells cultured in ABC medium in non-adhesive bacterial plates acquired a spheroid shape (200 ± 25 µm in diameter) four days after culture (Fig. 5d). The percentage of MESP1^+^ cells was retained in the suspension culture condition and >10^8^ cells were generated after 4 passages (Fig. 5e,f). Expansion of cells at passage 4 showed greater than 4-fold cell expansion (Fig. 5g).

### Assessment of differentiation potential into main cardiac lineages

**Figure 6:**
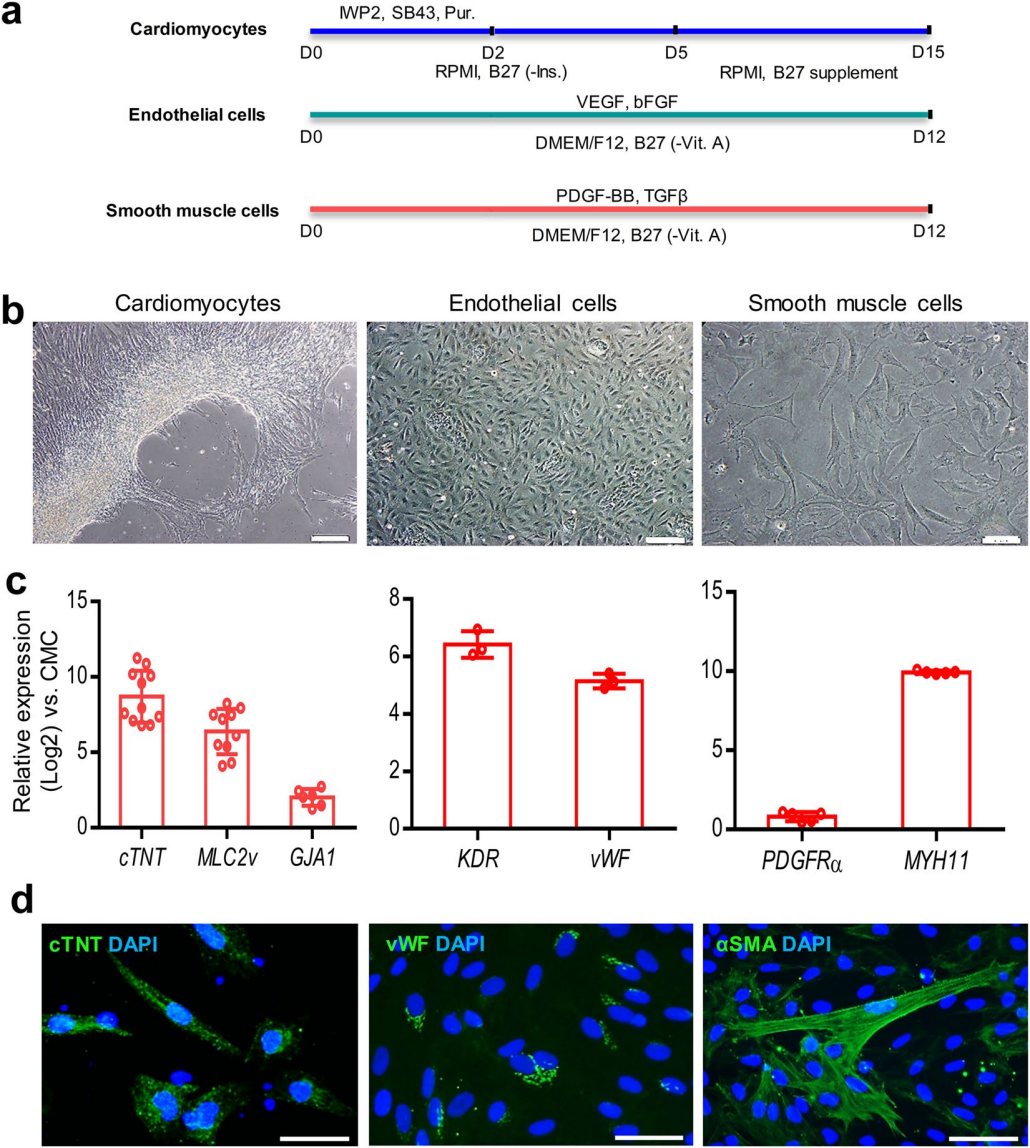
*In vitro* differentiation potential of adherently expanded CMCs in ABC medium. (**a**) Schematic illustration of induction protocols for differentiation of passage 10 CMCs into cardiomyocytes, endothelial, and smooth muscle cells. D: Day; SB43: SB431542; Pur: Purmorphamine; -Ins: Minus insulin; -Vit. A: Minus vitamin A. (**b**) Morphology of induced-CMCs into cardiomyocytes, endothelial and smooth muscle cells. Scale bars: 200 µm. (**c**) Expression analyses of cardiomyocytes (*cTNT*, *MLC2v*, and *GJA1*), endothelial (*KDR* and *vWF*), and smooth muscle (*PDGFRa* and *MYH11*) cell specific genes compared to undifferentiated CMCs. All data: mean ± SD. (**d**) Immunofluorescence staining of cTNT, vWF, and α-SMA. Cells were counter-stained with DAPI. Scale bars: 100 µm.

The differentiation potential of P10 CMCs into cardiomyocytes was examined (Fig. 6a)^18^. The morphology of CMCs changed into elongated cells along with substantial upregulation of the cardiomyocytes’ structural genes (*cTNT*, *MLC2v*, and *GJA1*) 15 days post-induction (Fig. 6b,c). Moreover, differentiated cells were positive for cTNT according to the results of immunofluorescence staining (Fig. 6d).

P10 CMCs were also induced into endothelial and smooth muscle cells (Fig. 6a)^19^. The cells acquired cobblestone-like morphology of endothelial and spindle shape of smooth muscle cells after induction (Fig. 6b). These differentiated cells substantially upregulated endothelial (*KDR* and *vWF*) and smooth muscle (*PDGFRa* and *MYH11*) markers 12 days post-differentiation (Fig. 6c). Immunofluorescence staining showed the presence of vWF and α-SMA positive cells (Fig. 6d).

### *In vivo* evaluation of CMCs

**Figure 7:**
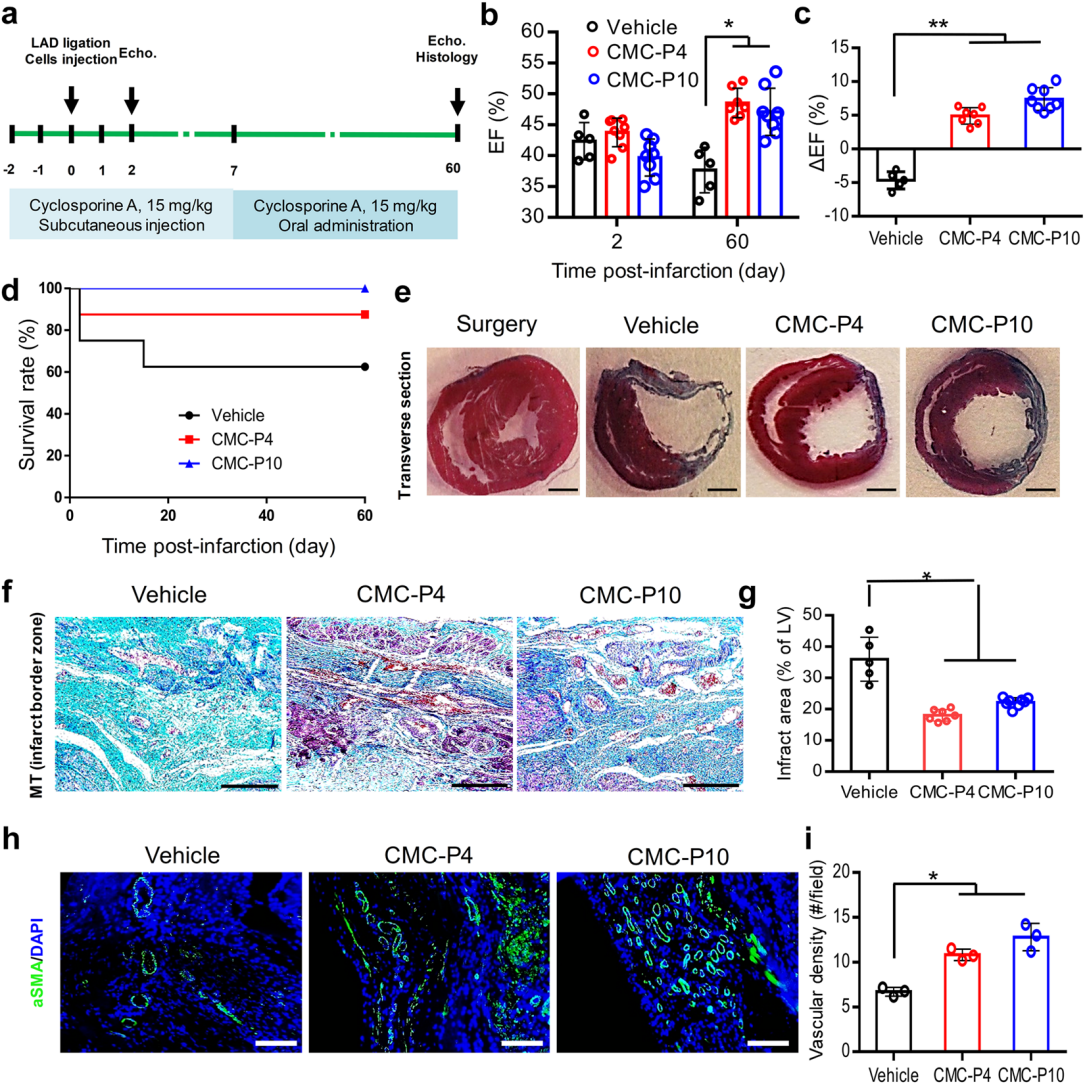
Cardio-protective effect of adherently expanded CMCs in ABC medium after transplantation into rat infarcted myocardium. (**a**) Schematic outline of the *in vivo* experiment. The animals received intramyocardial injections of the cells into the infarct border zone, 20 min after LAD artery ligation. Animals were immunosuppressed by the administration of cyclosporine A. Echo: Echocardiography. (**b**) The ejection fraction (EF) index of heart performance as evaluated by Echo at 2 and 60 days post-infarction. The data showed a significant increase of EF in the cell injected groups compared to the vehicle group. (**c**) The ΔEF represents the difference between the EF values measured at days 2 and 60 post-infarction for the *in vivo* groups. (**d**) Kaplan-Meier survival rate analysis of different *in vivo* groups. The cell transplanted groups had a higher survival rate compared to the vehicle group. (**e**) Masson’s trichrome (MT) stained transverse sections of rats’ hearts 60 days after infarction. Scale bar: 2 mm. (**f**) Cardiac fibrotic tissue stained with MT at the infarct border zones. Scale bars: 200 µm. (**g**) The measured infarct fibrotic area (%) stained by MT indicated significantly decreased scar tissues in the cell transplanted groups compared to the vehicle group. (**h**) Representative immunostaining against vascular structures with anti-αSMA. (**i**) The quantification indicated that the significant higher vascular densities in cell transplanted groups compared to vehicle group. Scale bars: 200 µm. LV: Left ventricle. All data: mean ± SD and statistically analyzed by one-way ANOVA followed by Tukey’s post-hoc test. *P ≤ 0.05; **P ≤ 0.01.

The safety and cardiac repair potential of cultured CMCs was examined *in vivo*. The adult rats received intramyocardial (IM) injections of 4 × 10^6^ P4 or P10 CMCs (CMC-P4 or CMC-P10) 20 min after MI induction (Fig. 7a). The animals were immunosuppressed with cyclosporine A. Their cardiac function was assessed by echocardiography. Heart performance, as assessed by ejection fraction (EF) and ΔEF, was substantially improved two months after cell transplantation compared to the vehicle group (Fig. 7b,c). At day 60 of the follow up period, the transplanted groups had a better survival rate in comparison to the vehicle group (Fig. 7d). Masson’s trichrome (MT) staining of the cardiac tissues harvested from the transplanted animals showed significantly reduced scar areas compared to the vehicle receiving group (Fig. 7e,f). The scar size of the left ventricle was significantly reduced in the CMC-P4 (18.0 ± 1.9%) and CMC-P10 (22.2 ± 1.5%) transplanted groups compared to the vehicle group (35.9 ± 7.0%; Fig. 7g).

Moreover, there were significantly more αSMA^+^ vascular structures in the transplanted groups compared with the vehicle group (Fig. 7h,i). No sign of teratoma formation was observed in transplanted hearts 60 days after injection.

## Discussion

In this study, initially, CMCs were generated by induction of three hPSC lines using a two-day differentiation protocol based on a small molecule working as a GSK3 inhibitor (CHIR)^12^. CMCs exhibited the molecular signature of early CPCs (co-expression of early and late cardiac transcription factors)^5,9,19,20^. This co-expressed panel of genes might reflect the transition and commitment of cells from mesoderm into an early cardiovascular fate. Although it has been reported that MESP1^+^ progenitor cells have the ability to differentiate into both cardiac and non-cardiac (other mesodermal) lineages^21^, expression of cardiac-restricted transcription factors such as *NKX2.5*, *ISL1*, and *MEF2c* might preclude the possibility of differentiation into non-cardiac cells in our culture system.

Based on the performed *in vitro* and *in vivo* developmental studies, it is accepted that *MESP1* expression is transient in the early stages of heart development and downregulated through progress of cardiogenesis^15,22–25^, which is consistent with the results obtained from our *in vitro* studies on hPSC-derived cardiovascular derivatives^12,18^. In order to develop a culture condition to expand and maintain the cells in the early stage of cardiovascular progenitors, we screened a potentially efficient pool of signaling pathway factors from a list of literature-based factors introduced as putative proliferative molecules^19,26–31^. Our screening resulted in the selection of A83-01, bFGF, and CHIR. Other factors as single or in combination, did not lead to expansion of CMCs. A83-01 is a potent inhibitor of TGF-β type I receptor ALK5, nodal receptor ALK7, and activin/nodal receptor ALK4^32^. A83-01 can potentially be used alone or in combination with other signaling molecules for expansion of CPCs^19,26,33^. bFGF, a member of the fibroblast growth factor (FGF) family, is a potent mitotic factor for a wide range of cell types, including CPCs^27,34^. Other FGF members such as FGF8 and FGF10 have been introduced as proliferation inducing factors in distinct CPC populations^5^. Of note, FGF ligands exert their roles through activation of PI3K/Akt or MAPK/ERK signaling pathways that interact downstream of other signaling pathways^5,35^. CHIR activates the Wnt pathway by inhibiting GSK3 signaling^18^. Multiple studies have shown a decisive role of the Wnt signaling pathway during embryo development, particularly cardiogenesis^5,36^. Therefore, a number of experiments have targeted this signaling pathway to induce expansion and improve differentiation of CPCs^19,37,38^. Interestingly, Wnt, TGF-β, and PI3K/Akt signaling pathways were among the significantly enriched pathways in MESP1^+^ cells^14,39^.

A proportion of cultured CMCs expressed the surface marker CD56, a proposed marker for mesodermal progenitors^40,41^. We have also observed expression of another surface marker in expanded CMCs, PDGFRα, which is a well-known CMC marker similar to MESP1^42^. Co-expression of PDGFRα and KDR has been previously used to identify the early CPCs^43,44^. However, the expression of KDR was downregulated in CMCs cultured in ABC medium after passaging. Consistently, a microarray result unveiled the enrichment of *PDGFRa*, but not *KDR*, in MESP1^+^ cells derived from hESCs. Flow cytometry analysis showed co-expression of PDGFRα and MESP1 in approximately 70% of cells^14^. In a similar study, flow cytometry results showed rare expression of KDR (5–7% of the population) in SSEA1^+^MESP1^+^ cells derived from differentiation of hPSCs^9^. Of note, there might be different subpopulations in cultured CMCs as we did not perform clonal assessments or cell sorting for cell populations resulting in high purity. Further studies are required to generate more homogeneous populations.

CMCs were expanded for approximately 11-fold at the early passages in ABC medium. This rate showed an insignificant, partial decrease in the late passages. The expansion rate, PDT, and total count of expanded cells in ABC medium were comparable with the results reported by Cao *et al*.^19^. Similarly, we observed a consistent cardiac gene expression profile and no detectable expression levels for the specific endoderm and ectoderm lineage markers in the CMCs during passaging^19^. Although this would indicate that the expanded CMCs had distinguishable gene expression signature from other cell types, a genome-wide gene expression assessment is required to provide a conclusive view in this regard.

As another characteristic of expanded CMCs in ABC medium, their *in vitro* differentiation capacity was evaluated; however, our cardiomyocyte differentiation protocol could not efficiently generate spontaneously beating cardiomyocytes; therefore, this suboptimal protocol should be further optimized. Possibly, more specific and directed differentiation protocols are required to mimic the signals generated from the cardiac niche that result in beating cardiomyocytes in order to provide the specific proofs and evidences for cardiomyogenic differentiation potential of expanded CMCs. Consistently, the expandable CPCs generated from transdifferentiation of mouse fibroblasts (named as induced CPCs; iCPCs) had differentiation potential into cardiovascular lineages as evidenced by fluorescent staining of specific markers; however, despite the existence of highly organized sarcomeres, the differentiated iCPCs into cardiomyocytes failed to spontaneously contract^20^.

Our *in vivo* results exhibited the safety and cardio-protection of injected CMCs in the heart with no signs of teratoma formation 60 days after injection. We showed that an IM injection of CMCs, regardless of passage number, may ameliorate heart performance 60 days post-infarction compared to the vehicle group. The improvement might be attributed to cardiac differentiation, paracrine effects, and increase in angiogenesis or cardio-protection in the acute infarct setting. The secretion of paracrine factors from CPCs has been shown^33,45^. In addition, alternative mechanisms could be post-MI inflammation regulation and indirect modulation of the natural immune responses to dying cells^46^. However, further experiments must be performed to determine the therapeutic mechanism and efficiency of CMC transplantation and CMC specific effects. Moreover, whether these mechanisms can mediate long-term repair or protection remains an open issue.

Furthermore, we established a simple, efficient protocol for long-term storage and suspension culture of CMCs to facilitate translation of our culture strategy into large-scale industrial applications. In order to increase the survival rate of CMCs after cryopreservation, we have used a potent anti-apoptotic small molecule (Rho kinase inhibitor; ROCKi, Y-27632). This is widely used, not only to circumvent the apoptosis process, but also to enhance the attachment of cells to their culture substrates^34,47^. We also benefited from ROCKi to improve the survival of CMCs after singularization for culture, cryopreservation, and transplantation. Another important finding of this study was the carrier-free static suspension culture and expansion of CMCs. To the best of our knowledge, this is the first report which explains the suspension culture system for expansion of hPSC-derived cardiovascular progenitors. This system facilitates the expansion of hPSC-CMC in fully controlled bioreactor systems for regenerative cell manufacturing. A study by Jha *et al*. showed higher proliferative characteristics of hPSC-derived CPCs under three-dimensional conditions stimulated by microgravity^48^.

In conclusion, we have introduced a culture medium that contains a combination of three signaling factors with the capability to maintain hPSC-CMCs in their proliferative progenitor state. The expanded CMCs retained the typical features of early cardiovascular progenitors with no safety concerns for tumorigenicity and might improve cardiac performance after transplantation into the infarct border zone. Moreover, they possessed the capacity for suspension culture and could be easily cryopreserved. These properties would make them suitable for use as raw materials in various applications such as cardiac cell therapy and tissue engineering. Two limitations of this study are: (1) the establishment of optimal induction protocols to efficiently differentiate expanded CMCs, *in vitro* and *in vivo* and (2) whether the regenerative capacity of CMCs is mediated through specific mechanisms for long-term repair or protection, which remains to be explored.

## Methods

### hPSC culture and CMC generation

Two hESC lines, RH5 and RH6, and an iPS line^49^ were cultured and expanded in suspension culture according to a previously described protocol^34^. Briefly, 2 × 10^5^ viable single cells/ml in hPSC medium were transferred to non-adhesive bacterial plates. The medium was conditioned an overnight on human foreskin fibroblasts which were inactivated by mitomycin C (Sigma-Aldrich, M0503). The hPSC medium contained DMEM/F12 (Gibco, 31331028) supplemented with 20% knockout serum replacement (KO-SR; Gibco, 10828-028), 1% nonessential amino-acids (NEAA; Gibco, 11140-035), 1% penicillin/streptomycin (Gibco, 15070-063), 1% insulin-transferrin-selenite (ITS; Gibco, 51500-056), 0.1 mM β-mercaptoethanol (Sigma-Aldrich, M7522), and freshly added 100 ng/ml bFGF (Royan Biotech). Cells were treated with 10 µM of ROCKi (Sigma-Aldrich, Y0503) for the first two days and the medium was refreshed every two days. In order to induce hPSCs into CMCs, 5-day old spheroids (175–200 µm in diameter) were treated with 12 µM of the small molecule, CHIR (Stemgent, 04-0004-10), in basal differentiation medium that consisted of RPMI 1640 (Gibco, 52400-041) supplemented with 2% B-27 without insulin (Gibco, A18956-01), 2 mM L-glutamine (Gibco, 25030-024), 1% NEAA, 1% penicillin/streptomycin, and 0.1 mM β-mercaptoethanol for one day followed by one-day culture in basal differentiation medium without small molecule^12^.

### Culture and maintenance of CMC

CMC spheroids were dissociated into single cells by treatment with Accutase solution (Sigma-Aldrich, A6964) for 3 min at 37 °C and subsequently cultured at a cell density of 5 × 10^4^ cells/cm^2^ on Matrigel-coated plates in basal maintenance medium that consisted of DMEM/F12 supplemented with 2% B-27 without vitamin A (Gibco, 12587-010), 2 mM L-glutamine, 1% NEAA, 0.1 mM β-mercaptoethanol, and freshly added chosen factors, 0.5 µM A83-01 (Stemgent, 04-0014), 100 ng/ml bFGF, and 3 µM CHIR, which we named ABC medium. Cells were treated overnight with 10 µM ROCKi and the medium was refreshed daily. CMCs were routinely passaged every 4 days by using Accutase and cultured on Matrigel-coated plates at a cell density of 5 × 10^4^ cells/cm^2^ in ABC medium.

### CMC characterization

For detailed methods on CMC characterization, see supplemental information.

### *In vivo* assessment of CMC

#### Acute Myocardial infarction (AMI) model

AMI was induced in adult male Wistar rats (weight: 250–300 g) according to a previously published protocol with some modifications^6^. Animals were housed and cared according to protocols approved by the Royan Institutional Animal Care and Use Ethical Committee in conformity with the NIH Guide for the Care and Use of Laboratory Animals. Immunosuppression was performed by subcutaneous (SC) administration of 15 mg/kg/day cyclosporine A (Novartis Pharma AG) two days before surgery to seven days post-MI. During the remainder of the experiment, the animals received 15 mg/kg/day cyclosporine A added to their drinking water. Prior to surgery, the animals were anesthetized with intraperitoneal (IP) injections of 0.1 mg/kg Medetomidine (Syva, Spain) and 75 mg/kg Ketamine (Alfasan, The Netherlands). Once anesthetized, the animals were intubated and mechanically ventilated (Harvard, USA) with a mixture of room air, oxygen, and 1% Isoflurane to maintain a deep level of anesthesia. The chest area was shaved and the surgical site was prepared via aseptic techniques. A left thoracotomy was performed to expose the left ventricle and infarction was achieved by permanent ligation of the left anterior descending artery (LAD) with 6-0 monofilament polypropylene suture material (Keyhan Teb, Iran). The AMI was established by regional color change of the myocardial surface. In this study, 30 rats were divided into four experimental groups: sham-operated (surgery; n = 6), control AMI-induced (vehicle; n = 8), and treatment (CMC-P4; n = 8 and CMC-P10; n = 8). With the exception of the sham group animals, the other rats received either 500 µl PBS/10%BSA in the vehicle group or 4 × 10^6^ ROCKi treated-cells in 500 µl PBS/10%BSA in the treatment groups. IM injections (100 µl per injection) were administered at five sites of the infarct border zone 20 min after the LAD ligation. After the injection, the ribs, muscle layers, SC layer, and skin were closed and the animals received an IP injection of 1 mg/kg Atipamezole (Syva, Spain). The animals were placed on a heating stage until recovery. They received a prophylactic antibiotic (Enrofloxacin, 15 mg/kg, SC, Rooyan Darou, Iran) and analgesic (Tramadol, 20 mg/kg, SC, Darou Pakhsh, Iran) for three days to prevent infection and to perform pain management, respectively. At the end of the study, rats were euthanized using CO_2_ inhalation.

#### Echocardiographic assessment

Cardiac function was evaluated in rats by echocardiographic assessment using an ultrasound system (Mindray, USA) on days 2 (baseline) and 60 after the AMI. The animals were anesthetized by IP injections of Medetomidine and Ketamine, and their chests were shaved. An experienced cardiologist blinded to the treatment groups performed the ultrasound. The data was acquired as two-dimensional images. End-diastolic and systolic volumes were obtained to assess the left ventricular EF. ΔEF for each animal was calculated by subtracting the EF value of baseline from EF value on day 60.

#### Histological studies

Two months after surgery, the animals were sacrificed and their hearts were removed and fixed with 10% formalin at room temperature (RT) for 72 h. The processed hearts were embedded in paraffin and cut into 5 µm thick sections. After deparaffinization and hydration, the heart sections were stained by MT to visualize the infarct area. The percentage of the infarct area was calculated with ImageJ software. We chose six slides for each animal and calculated the mean percentage of the infarct area.

For immunofluorescence staining, deparaffinized and rehydrated sections underwent antigen retrieval using Target Retrieval Solution, pH 9, (Dako, S2368) for 20 min. Thereafter, sections were permeabilized with 0.5% triton X100 at RT for 30 min, blocked for 1 h at 37 °C and then incubated overnight with primary antibodies at 4 °C. The next day, sections were washed and treated with secondary antibodies at 37 °C for 1 h, counterstained with DAPI and observed under fluorescence microscope (Olympus; IX71). To measure vascular density in infarcted regions, the number of a-SMA^+^ vascular structures was counted in five fields per section and three sections were utilized for each animal^6^.

### Freeze-thaw procedure

For long-term storage of CMCs, we used Accutase to dissociate the spheroids into single cells. After centrifugation at 1500 rpm for 5 min, 2 × 10^6^ cells were dissolved in 500 µL cold freezing medium that contained 90% FBS, 10% DMSO, and 10 µM ROCKi. Cells were kept overnight in a −80 °C freezer and transferred to a liquid nitrogen tank the next day. In order to recover the cryopreserved cells, the frozen vials were held in a 37 °C water bath until most of the ice thawed. Fresh medium that contained 10 µM ROCKi was added to cells and, after centrifugation, the cells were resuspended in ABC medium that contained 10 µM ROCKi and cultured on Matrigel-coated plates. Trypan blue exclusion method was used to calculate cell viability as previously described^12^.

### Suspension culture

In this experiment, single CMCs were cultured at a density of 2 × 10^5^ cells/ml in ABC medium that contained 10 µM ROCKi in non-adhesive bacterial plates. The medium was refreshed 2 days later with fresh ABC medium without ROCKi and renewed every two days. Spheroids generated from the expanded CMCs, with 200–250 µm diameter, were passaged.

### Statistical analysis

All data are presented as median or mean ± standard deviation (SD) (obtained from at least three independent replicates). After testing the normal distribution of data, the significance of differences were statistically analyzed by SPSS 16.0 (SPSS, Inc.) using the unpaired t-test or one-way ANOVA followed by Tukey’s post-hoc test. A P-value of ≤ 0.05 was considered to be statistically significant.

## Supplementary information

Supplementary information

## Data Availability

The datasets generated during and/or analysed during the current study are available from the corresponding author on reasonable request.

## Supplementary information

is available this paper at 10.1038/s41598-019-52516-8.
