# Supplementary material for: Expansion of Human Pluripotent Stem Cell-derived Early Cardiovascular Progenitor Cells by a Cocktail of Signaling Factors
Source: Sci Rep. 2019 Nov 5;9:16006. doi: 10.1038/s41598-019-52516-8 (PMC6831601; doi:10.1038/s41598-019-52516-8)
Supplement: Supplementary file 1 — Supplementary information [file 41598_2019_52516_MOESM1_ESM.pdf]

Supplemental information

Expansion of Human Pluripotent Stem Cell-derived Early Cardiovascular Progenitor Cells by a Cocktail of Signaling Factors

Sadaf Vahdat, Sara Pahlavan, Elena Mahmoudi, Maryam Barekat, Hassan Ansari, Behnaz Bakhshandeh, Nasser Aghdami, Hossein Baharvand*

1. Supplementary methods

1.1. Immunofluorescence staining and flow cytometry

Cells were fixed with 1% paraformaldehyde for 20 min at room temperature (RT) followed by incubation with ice-cold 90% methanol (Merck, 106007) at 4°C for 15 min. After washing twice with PBS/0.5% BSA, the cells were incubated overnight with primary antibodies in PBS/0.5% BSA/0.1% Triton X-100 (Sigma, x100) at 4°C. The cells were then washed twice with PBS/0.5% BSA the next day and treated with secondary antibodies in PBS/0.5% BSA/0.1% Triton X-100 for 1 h at RT. For flow cytometry, the cells were analyzed with a BD FACSCalibur system (BD Biosciences, San Jose, CA, USA). Data analysis was performed with Flowing software 2.5 (Turku Centre for Biotechnology). Immunofluorescence stained cells were observed by a fluorescent microscope (Olympus, IX71, Japan). The primary and secondary antibodies used for flow cytometry and immunofluorescence staining were: anti-MESP1 (Abcam, Ab77013), anti-Ki67 (BD Biosciences, 561165 and Abcam, Ab92742), anti-PDGFRa (Santa Cruz, SC21789), anti-CD56 (BD Biosciences, 555516), anti-cTNT (Thermo Scientific, MA5-12960), anti-vWF (Abcam, Ab68545), anti-α-SMA (Abcam, Ab7817), Alexa488-conjugated donkey anti-mouse secondary antibody (Invitrogen, a21202), Alexa546-conjugated donkey anti-rabbit secondary antibody (Invitrogen, a10040), and Alexa546-conjugated donkey anti-mouse secondary antibody (Invitrogen, a10036).

1.2. Quantitative real-time RT-PCR

For molecular analysis of gene transcription, total RNA was isolated manually using TRIzol reagent (Invitrogen, 15596-026). Potential DNA contamination was avoided by using a DNase1 Kit (Fermentas, en0521) according to the manufacturer's instructions. First strand cDNA synthesis was performed using 2 µg of total RNA with a PrimeScript™ RT Reagent Kit (Perfect Real Time, TaKaRa, RR037A), and real-time PCR was carried out using an SYBR Premix Ex Taq Kit (TaKaRa RR041A) with a Rotor Gene Corbett System (R080873). Relative gene expression was calculated by the $2^{-\Delta\Delta ct}$ formula. *GAPDH* was the housekeeping gene. Supplementary Table S1 lists the primer information.

1.3. Population doubling time (PDT) measurement

Passages 4 (P4) and 10 (P10) CMCs were seeded at a density of 5×10^4 cells/cm^2 on Matrigel-coated 4-well multiwell plates in ABC medium. PDT was measured as previously described [1]. Trypan blue exclusion method was used to determine the cell counts at 2, 3, and 4 days after culture. The following formula was used to calculate PDT:

$N = N_0 2^{t/T}$

Where:

N = Daily cell count

N_0 = Initial cell count

T = PDT.

1.4. Teratoma formation assay

Tumorigenicity of the expanded CMCs at P0, P4, and P10 were examined by subcutaneous (SC) injections of 5×10^6 cells in 100 µl of DMEM/F12:Matrigel (1:1) and 10 µM ROCKi in

8-week old male Nude mice (B6NU). A total of 5×10^6 hPSCs were injected as the positive control. The mice were maintained for 3 months, after which they were euthanized using CO_2 inhalation and examined for the presence of teratoma formation. All experimental procedures and maintenance of the mice were conducted according to protocols approved by Royan Institute's Animal Care and Use Ethical Committee in conformance with the NIH Guide for the Care and Use of Laboratory Animals.

1.5. Directed differentiation into major cardiovascular lineages

The expanded CMCs were subjected to cardiomyocyte differentiation. These cells were seeded at a density of 4×10^5 cells/cm^2 on Matrigel-coated plates in basal differentiation medium supplemented with 5 µM IWP2, 5 µM purmorphamine, and 5 µM SB431542 for 2 days. Thereafter, the cells were washed once with DPBS (Gibco, 21600-010) and further cultured in basal differentiation medium without small molecules. The medium was renewed every 3 days. In order to generate endothelial or smooth muscle cells, the CMCs were seeded at a density of 1×10^4 cells/cm^2 on Matrigel-coated plates in basal maintenance medium supplemented with 50 ng/ml VEGF and 10 ng/ml bFGF (for endothelial cells) or 10 ng/ml PDGF-BB and 2 ng/ml TGF-β1 (for smooth muscle cells) for 12 days according to a published protocol with slight modifications [2]. The medium was refreshed every 3 days.

Supplementary Tables

Supplementary Table S1. Genes and primer sequences used for quantitative reverse transcriptase-PCR.

Gene name	Primer sequences (5'-3')	
MESP1	F: ACCTTCGAAGTGGTTCCTTG	R: TCCTGCTTGCCTCAAAGTGT
SSEA1	F: CTTCAACTGGACGCTCTCCTA	R: GTTGGTGGTAGTAGCGGACC
ISL1	F: TACAAAGTTACCAGCCACC	R: GGAAGTTGAGAGGACATTGA
PDGFRα	F: TACACTTGCTATTACAACCACA	R: ATCCTCCACGATGACTAAAT
KDR	F: CCAGCCAAGCTGTCTCAGT	R: CTGCATGTCAGGTTGCAAAG
NKX2.5	F: TCTATCCACGTGCCTACAG	R: CCTCTGTCTTCTCCAGCTC
MEF2c	F: TCCGAGTTCTTATTCCACC	R: ATCCTCCCATTCCTTGTC
SOX17	F: CGGTATATTACTGCAACTATCCTG	R: GGATTTCCTTAGCTCCTCCA
AFP	F: AAATGCGTTTCTCGTTGCTT	R: GCCACAGGCCAATAGTTTGT
PAX6	F: GTCCATCTTTGCTTGGGAAA	R: TAGCCAGGTTGCGAAGAACT
TUBB3	F: GTATCCCGACCGCATCAT	R: TCTCATCCGTGTTCTCCA
OLIG2	F: CGACTCATCTTTCCTTCTCTAA	R: CGCACTTACCTCATCATTG
GFAP	F: GAGATGCGGGATGGAGAG	R: TAGGGACAGAGGAGGGAG
cTNT	F: ATGATGCATTTTGGGGGTTA	R: CAGCACCTTCCTCCTCTCAG
MLC2v	F: CTTGGGCGAGTGAACGT	R: CTGGTCAACCTCCTCCTTG
GJA1	F: GCTATGACAAGTCTTTCCCA	R: CAGTTTCTCTTCCTTTCGCA
vWF	F: CATTCAGCTAAGAGGAGGAC	R: TTGTGTTCATCAAAGGGTGG
GAPDH	F: CTCATTTCCTGGTATGACAACGA	R: CTTCCTCTTGTGCTCTTGCT

Supplementary Table S2. Tumorigenicity assessment of expanded CMCs in ABC medium.

Cell source	Cell # (× 10^6)	Time to visualization of mass (days)	Mice with teratoma/total mice	Period (days)
CMC-*P0	5	-	0/3	90
CMC-P4	5	-	0/3	90
CMC-P10	5	-	0/3	90

*P: Passage

2. Supplementary figures

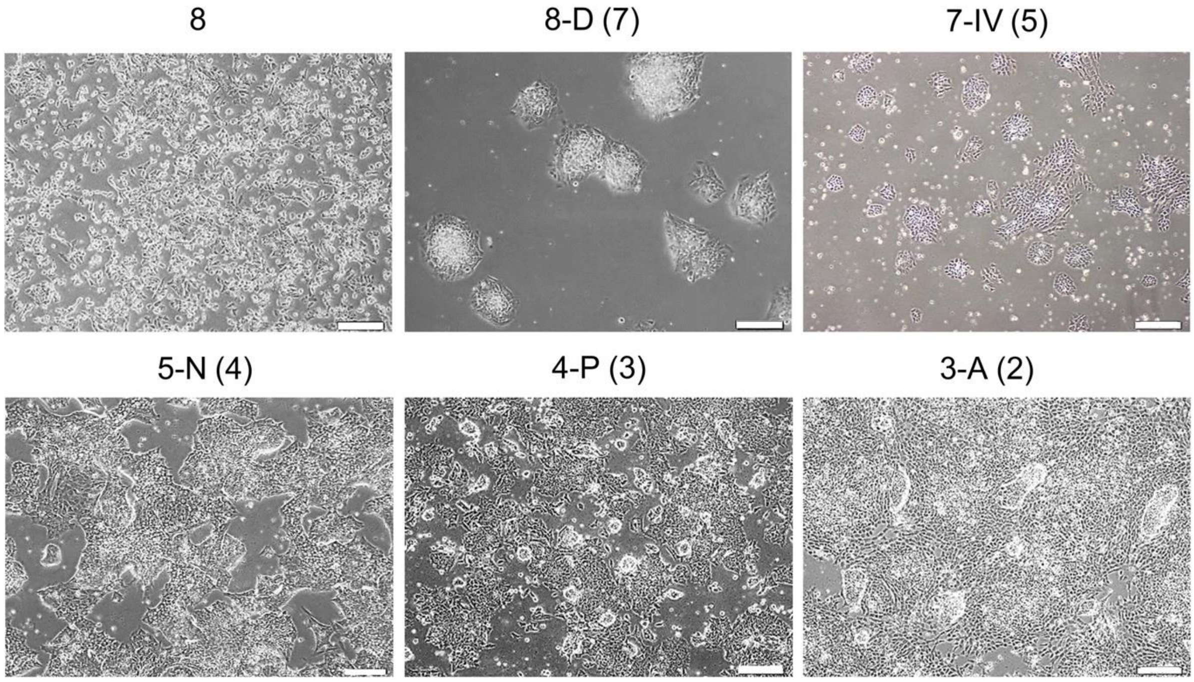

Supplementary Figure S1. Phase contrast images show the morphology of RH5-derived CMCs in different combinations of the candidate signaling factors 2 days after adherent culture. Serial removal of signaling factors from the initial cocktail of 8 factors (8) resulted in media that contained different combinations of the signaling factors (7, 5, 4, 3 and 2). Scale bars: 200 µm. A: A83-01; D: Dorsomorphine; I: IGF1; N: NRG1 agonist; P: Pioglitazone; V: Vitamin C.

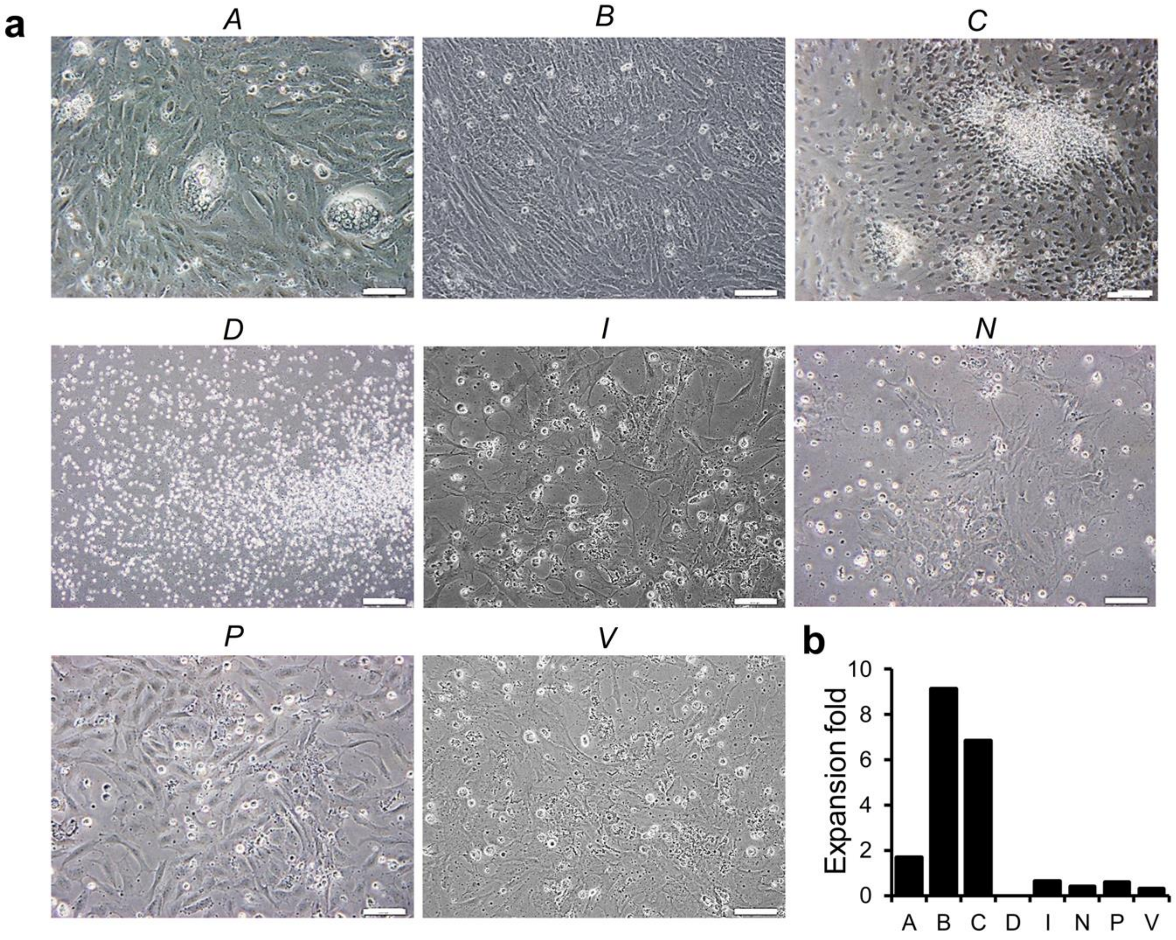

Supplementary Figure S2. Adherent culture of RH5-derived CMCs in medium that contained only one chemical. (a) Phase contrast images show morphology of the cultured CMCs. Scale bars: 200 µm. (b) Fold-change of expansion of CMCs cultured in medium that contained only one chemical. A: A83-01; B: bFGF; C: CHIR99021; D: Dorsomorphine; I: IGF1; N: NRG1 agonist; P: Pioglitazone; V: Vitamin C.

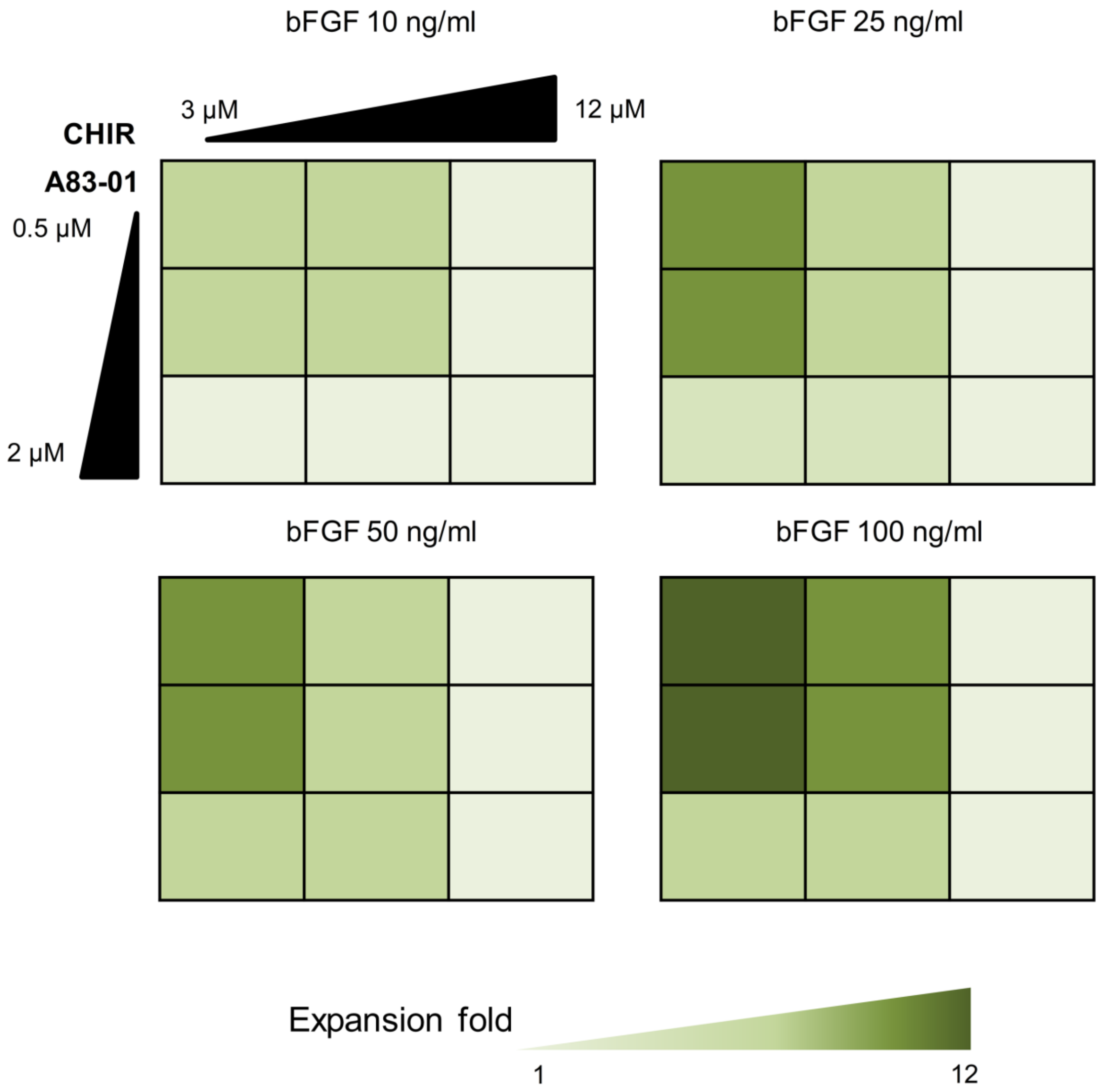

Supplementary Figure S3. Schematic representation of the concentration-dependent expansion rate of CMCs in different doses of A83-01 (0.5, 1, and 2 μM), bFGF (10, 25, 50, and 100 ng/ml) and CHIR (3, 6, and 12 μM). We selected 0.5 μM A83-01, 100 ng/ml bFGF, and 3 μM CHIR for adherent culture and maintenance of CMCs.

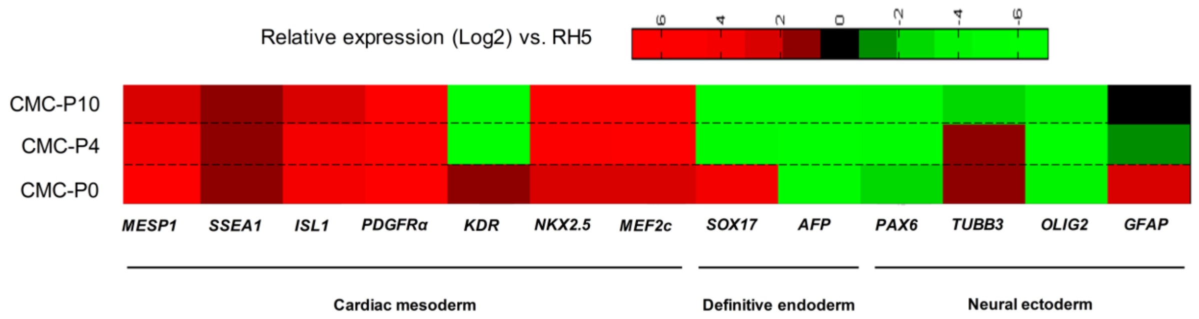

Supplementary Figure S4. Heatmap illustration of CMC gene expression pattern after passaging in the adherent culture condition. Expressions of cardiac mesoderm, definitive endoderm, and neural ectoderm genes showed a similar pattern at different passages (P0, P4, and P10) of RH5-derived CMCs in ABC medium.

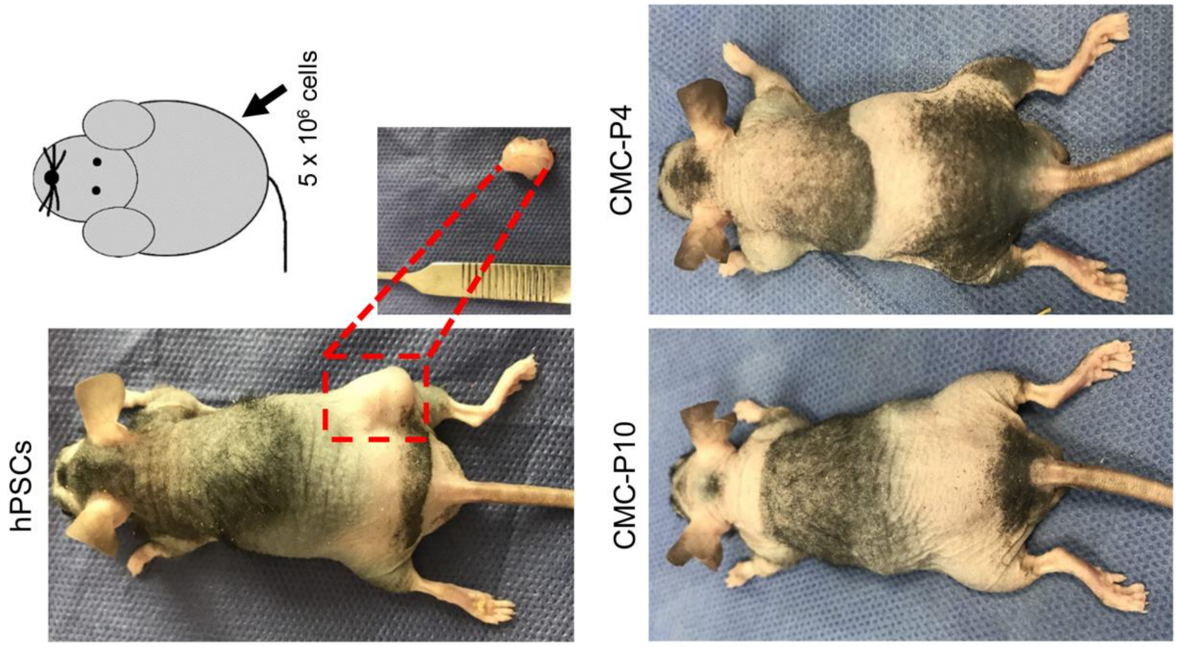

Supplementary Figure S5. Tumorigenicity assessment of adherently expanded CMCs. We subcutaneously injected 5 × 10^6 CMCs (passages 0, 4 or 10) or pluripotent cells into the right legs of 8-week old male Nude mice. After 90 days follow-up, we did not observe any signs of mass formation in the CMC injected groups at all assessed passages. Tumor formation was observed 20 days after injection of hPSCs in the mice.

References

[1] Vahdat S, Mousavi SA, Omrani G, Gholampour M, Sotoodehnejadnematalahi F, Ghazizadeh Z, et al. Cellular and molecular characterization of human cardiac stem cells reveals key features essential for their function and safety. Stem Cells Dev. 2015;24:1390-404.
[2] Cao N, Liang H, Huang J, Wang J, Chen Y, Chen Z, et al. Highly efficient induction and long-term maintenance of multipotent cardiovascular progenitors from human pluripotent stem cells under defined conditions. Cell research. 2013;23:1119-32.
